# Successful one‐stage operation for type B acute intramural hematoma with descending aortic rupture

**DOI:** 10.1002/ccr3.5267

**Published:** 2022-01-09

**Authors:** Akihisa Furuta, Hironobu Morimoto, Shogo Mukai, Daisuke Futagami, Junya Kitaura

**Affiliations:** ^1^ Department of Cardiovascular Surgery Fukuyama Cardiovascular Hospital Hiroshima Japan

**Keywords:** frozen elephant trunk technique, single‐stage operation, type B acute intramural hematoma

## Abstract

A 76‐year‐old man who complained of back pain was referred to our hospital. Computed tomography revealed an intramural hematoma with a descending aortic rupture. Total arch replacement with the frozen elephant trunk technique and thoracic endovascular aortic repair was performed emergently in one stage. The patient was discharged without symptoms.

## Graphical abstract

Total arch replacement with the frozen elephant trunk technique and subsequent thoracic endovascular aortic repair in one stage was a valuable method for treating descending aortic rupture complicated by type B acute intramural hematoma.

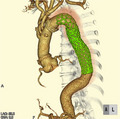

## 1 INTRODUCTION

Descending aortic rupture complicated by type B acute aortic dissection or intramural hematoma (IMH) is a rare and fatal condition with a mortality rate of more than 50%.^1^ Although emergent surgical treatment such as open surgery and/or thoracic endovascular aortic repair (TEVAR) is required in this condition, treatment is often associated with mortality and morbidity. Left thoracotomy may involve a great risk of mortality and major complications such as bleeding and paraplegia.^2^ Medial sternotomy provides common operative fields and facilitates ordinary procedures, such as ascending aorta perfusion. However, manipulations of the descending aorta are restricted, leading to mortality and morbidity. Therefore, additional treatment such as the frozen elephant trunk (FET) technique and/or TEVAR may be the key for better outcomes. TEVAR is a less‐invasive treatment; nevertheless, this procedure is sometimes restricted to some patients because of anatomical reasons and it also has a risk of leading to postoperative rupture.^3^

Herein, we describe a case of descending aortic rupture complicated by type B acute IMH in which open‐total arch replacement (TAR) using the FET technique followed by TEVAR was carried out in one stage.

## 2 CASE REPORT

**FIGURE 1:**
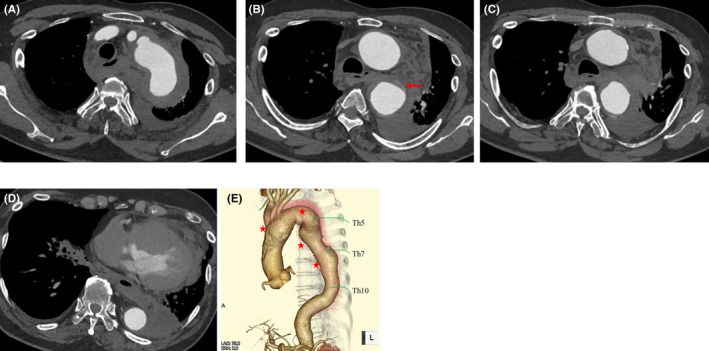
Preoperative computed tomography. Computed tomography shows type B acute intramural hematoma with rupture in the descending aorta. (A) 5th thoracic vertebra. Thrombosed false lumen of the aorta ranges from the arch to the descending aorta at the 7th thoracic vertebra. (B) 6th thoracic vertebra. The bleeding site seems to be located at the level of the 6th thoracic vertebra (arrow). (C) 7th thoracic vertebra. Hemothorax is observed in the left pleural cavity. The site of primary entry and ulcer‐like projections is unclear. (D) 10th thoracic vertebra. The descending aorta at the level of the 10th thoracic vertebra is normal in size and shape without dissection. (E) Three‐dimensional images reconstructed by computed tomography. Aortic aneurysms in the middle and distal aortic arch and descending aorta with diameters of 50 mm and 40 mm (four stars), respectively

A 76‐year‐old man with no significant medical history visited another hospital for back pain, where computed tomography (CT) suggested that he might have had an aortic rupture. The patient was urgently transferred to our hospital for treatment. During transfer, the patient lost consciousness due to decreased blood pressure. On arrival, he regained consciousness with a relatively low blood pressure of 80 mm Hg and a rapid heart rate of 94/min. CT revealed a type B acute IMH with rupture involving the descending aorta (Figure 1 and Video S1). The IMH extended from the distal arch after the bifurcation of the left subclavian artery to the descending aorta at the level of the 7th thoracic vertebra. The proximal arch at the bifurcation of the right brachiocephalic artery was dilated, with a diameter of 45mm. An aortic aneurysm was also detected in the middle and distal aortic arch, with a diameter of 50 mm, while the descending aorta had a diameter of 40 mm. A rupture occurred in the left pleural cavity, which seemed to be located at the level of the 6th thoracic vertebra, although the primary entry site and location of the ulcer‐like projection were unclear.

Because open‐lateral thoracotomy would involve a great risk of mortality and morbidity, and the size and configuration of the aorta would not provide a reasonable landing for TEVAR, TAR with FET was first considered as a surgical option. However, the longest commercial open stent‐graft would not be able to cover the descending aortic aneurysm and projected bleeding site, because this would require a graft length of at least 200 mm. Therefore, we decided to perform TAR with the FET technique followed by TEVAR in one stage.

Surgery was performed under general anesthesia using transesophageal echocardiography. As atherosclerosis was found in the ascending aorta, cardiopulmonary bypass was established by the right femoral and right brachiocephalic arteries and bicaval cannulation after a median sternotomy. After the body temperature reached 28°C, hypothermic circulatory arrest was initiated with antegrade cerebral perfusion through the brachiocephalic, left carotid, and subclavian arteries. An open stent‐graft (J Graft Frozenix, Japan Lifeline, Tokyo, Japan) with a diameter of 31 mm and a length of 12 cm was inserted into the distal arch and the distal site was anastomosed with a four‐branched graft with a diameter of 28 mm (J Graft, Japan Lifeline). After anastomosis of the neck vessels and the proximal site, cardiopulmonary bypass was terminated. A sheath for TEVAR was inserted via the exposed right femoral artery, and a stent‐graft with a diameter of 34 mm and a length of 15 cm (CTAG, W. L. Gore & Associates, DEL) was deployed in the descending aorta to overlap the 8‐cm length of the proximal side of the stent‐graft with the distal side of the open stent‐graft. Operative, cardiopulmonary bypass, aortic cross‐clamp, and circulatory arrest times were 359, 187, 97, and 42 min, respectively.

**FIGURE 2:**
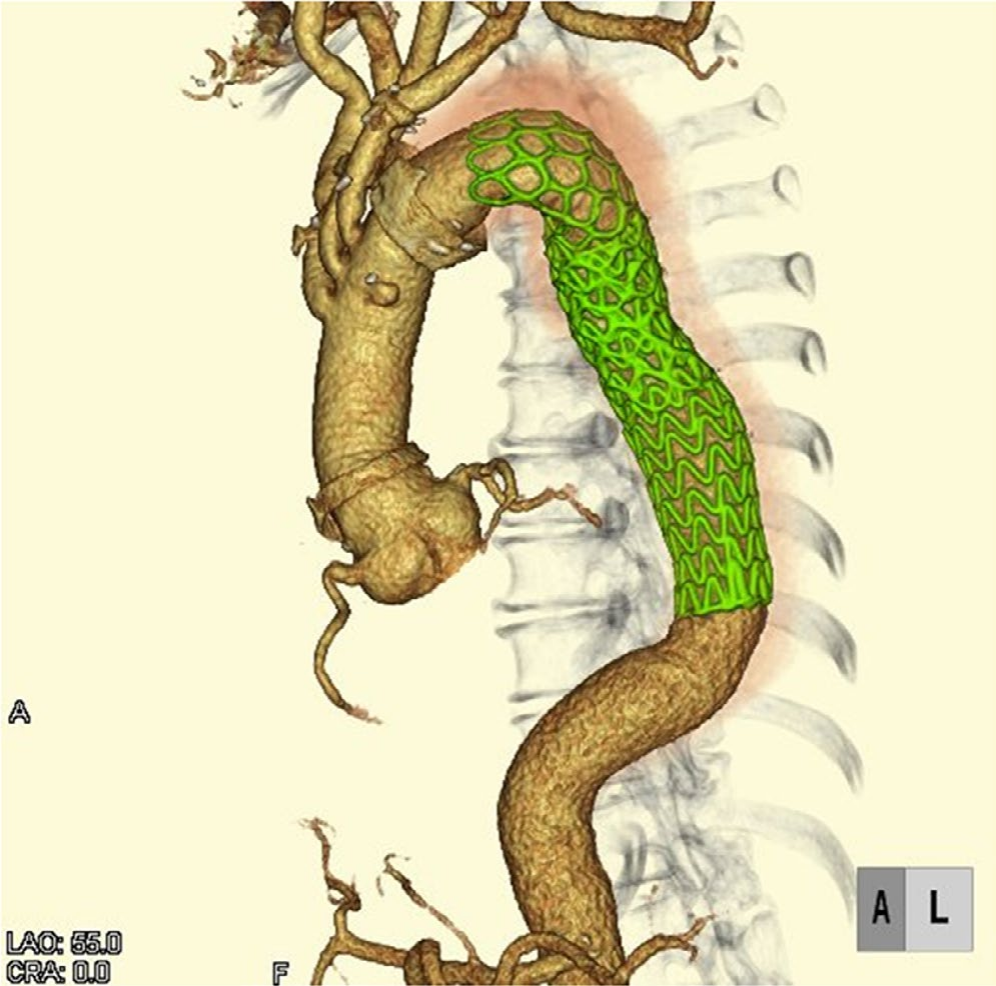
Postoperative computed tomography. Postoperative computed tomography demonstrates successful graft anastomosis and an appropriate position of the open stent‐graft and endovascular stent‐graft

In the postoperative period, although there were abnormal neurological signs, prolonged intubation was required for six days due to respiratory failure. Pseudomonas pneumonia and congestive failure were found on the 10th postoperative day, requiring additional medical treatment. Postoperative CT demonstrated successful graft anastomosis and stent‐graft position (Figure 2). The patient was discharged on the 28th postoperative day without symptoms.

## 3 DISCUSSION

IMH is a disease found on the acute aortic syndrome spectrum where hemorrhage occurs in the media of the aortic wall in the absence of demonstrative two‐lumen flow and primary entry. IMH is diagnosed if circular or crescentic thickening of the aortic wall >5 mm is present, in the absence of detectable blood flow in the vessel wall.^4^ Penetrating atherosclerotic ulcer refers to a focal lesion that ulcerates the intima and disrupts the internal elastic lamina of the aortic wall.^4^ Because of their visual similarities of these entities, differentiation of IMH from penetrating atherosclerotic ulcers is challenging. Nevertheless, penetrating atherosclerotic ulcers can often be identified as irregularities in the intimal layer, calcification of the ulcer edges, typical of atherosclerotic plaques, and a localized hematoma. We diagnosed this case as IMH because a relatively extended and crescentic hematoma and fewer atherosclerotic plaques were observed around the rupture site.

Rupture of the descending thoracic aorta complicated with acute aortic dissection or IMH requires an emergent surgical intervention because of the fatality of this condition.^1^ Options for surgical intervention for IMH with descending aortic rupture are divided into two types—open surgery and endovascular repair. Left thoracotomy carries a major risk of mortality and fatal complications. Minami et al. reported the clinical outcomes of emergency surgery acute type B aortic dissection with rupture, where the rate of early mortality was found to be 14%, and the rates of cerebral infarction and paraplegia were 29% and 7%, respectively. Another option is the replacement of the arch and descending aorta through median sternotomy with or without a small thoracotomy. This also carries a high risk of mortality and morbidity because of the technical difficulties associated with the manipulation of the descending aorta. The FET technique, which is an established strategy of employing a median sternotomy to address extensive aneurysms and dissection, permits single‐stage repair for extensive aortic diseases and promotes favorable aortic remodeling.^5^ As it also provides sufficient proximal landing and facilitates distal endovascular repair, this technique in combination with TEVAR can contribute to saving thoracotomy and reducing operative risk. TEVAR should be considered the first‐line treatment for complicated type B acute aortic dissection or IMH; however, in the case of aortic rupture, it gives rise to a potential risk of postoperative rupture and reinterventions if used in inappropriate circumstances.^1^, ^2^, ^3^ In our case, CT indicated type B acute IMH with rupture involving the descending aorta. The bleeding site was not explicitly shown by CT and aortic aneurysms were found in the aortic arch and descending aorta. Therefore, a long segment of the involved vessel, ranging from the ascending aorta to the descending aorta at the level of the 10th thoracic vertebra was treated. The size and configuration of the arch were not appropriate for TEVAR because of the potential risk of type Ia endoleak and subsequent rupture. Single‐TAR with the FET technique via median sternotomy could not cover the descending aortic aneurysm and the projected bleeding site, precluding a two‐stage surgery. Therefore, we decided to perform TAR with the FET technique via a median sternotomy combined with subsequent TEVAR in one stage.

Spinal cord ischemia is one of the major complications in descending aortic repair and the risk was reported to be 4%–7% after TEVAR and 2%–28% after open surgery.^6^ In our case, the Adamkiewicz artery was not identified on initial CT, and cerebrospinal fluid drainage for the prevention of spinal cord ischemia was not conducted due to the emergency. To avoid the complication of spinal cord ischemia, we maintained a reasonable mean blood pressure of >80 mm Hg during the acute postoperative phase and routinely evaluated the neurological condition. The patient was discharged on the 28th postoperative day without neurological deficits.

## 4 CONCLUSION

Total arch replacement with the FET technique and subsequent TEVAR in one stage was a valuable method for descending aortic rupture complicated with type B acute IMH where single‐open surgery or TEVAR was infeasible.

## CONFLICT OF INTEREST

The authors declare no conflicts of interest.

## AUTHOR CONTRIBUTIONS

Akihisa Furuta involved in study concept/design, drafting article and critical revision, approval of the article. Hironobu Morimoto involved in study concept/design, critical revision, and approval of the article. Shogo Mukai involved in study concept/design and critical revision. Daisuke Futagami involved in data interpretation and approval of the article. Junya Kitaura involved in critical revision and approval of the article.

## ETHICAL APPROVAL

Approval of the International Review Board was not required at our institution because this study was a case report.

## CONSENT

Written informed consent was obtained from the patient for publication of this report under the journal's patient consent policy.

## Supporting information

Supplementary Material

## Data Availability

The data that support the findings of this study are available from the corresponding author upon reasonable request.
